# Clade-Specific Alterations within the HIV-1 Capsid Protein with Implications for Nuclear Translocation

**DOI:** 10.3390/biom12050695

**Published:** 2022-05-12

**Authors:** Alexej Dick, Megan E. Meuser, Simon Cocklin

**Affiliations:** Department of Biochemistry & Molecular Biology, Drexel University College of Medicine, Rooms 10307, 10309, and 10315, 245 North 15th Street, Philadelphia, PA 19102, USA; megan.meuser@yale.edu

**Keywords:** HIV-1 capsid protein, assembly, nuclear import factor recognition, CPSF-6, NUP-153, surface plasmon resonance

## Abstract

The HIV-1 capsid (CA) protein has emerged as an attractive therapeutic target. However, all inhibitor designs and structural analyses for this essential HIV-1 protein have focused on the clade B HIV-1 (NL4-3) variant. This study creates, overproduces, purifies, and characterizes the CA proteins from clade A1, A2, B, C, and D isolates. These new CA constructs represent novel reagents that can be used in future CA-targeted inhibitor design and to investigate CA proteins’ structural and biochemical properties from genetically diverse HIV-1 subtypes. Moreover, we used surface plasmon resonance (SPR) spectrometry and computational modeling to examine inter-clade differences in CA assembly and binding of PF-74, CPSF-6, and NUP-153. Interestingly, we found that HIV-1 CA from clade A1 does not bind to NUP-153, suggesting that the import of CA core structures through the nuclear pore complex may be altered for viruses from this clade. Overall, we have demonstrated that in silico generated models of the HIV-1 CA protein from clades other than the prototypically used clade B have utility in understanding and predicting biology and antiviral drug design and mechanism of action.

## 1. Introduction

The HIV-1 capsid (CA) performs essential roles both early and late in the life cycle of HIV. The capsid is initially translated as the central region of the Gag polyprotein. As the virus buds, Gag is processed by the viral protease to produce three discrete new proteins—matrix protein (MA), CA, and nucleocapsid (NC)—as well as several smaller spacer peptides. After the capsid has been liberated by proteolytic processing, it rearranges into the conical core structure surrounding the viral genome at the center of the mature virus.

The HIV-1 capsid shell comprises about 250 CA hexamers and 12 CA pentamers, comprising about 1500 monomeric CA proteins in total. The multimers interact noncovalently to form the shell’s curved surface. CA itself is composed of two domains: the N-terminal domain (CA_NTD_) and the C-terminal domain (CA_CTD_). Several structures of the CA have been determined [1,2,3,4,5,6,7], with the most informative being those of the disulfide-engineered hexameric structure [8,9] and the more recent native hexameric structure [10].

The CA protein structure and stability are critical for uncoating, reverse transcription, nuclear entry, selection of the sites of integration, and assembly. Moreover, the capsid is also important for cloaking the DNA product from intracellular immune surveillance. To achieve these functions, HIV-1 CA interacts not only with itself but with host factors including TRIM5α [11,12,13,14,15,16,17,18,19,20,21,22], cleavage and polyadenylation specific factor 6 (CPSF6) [23,24,25,26,27,28], nucleoporins 153 and 358 (NUP153, NUP358) [25,26,28,29,30,31,32,33,34,35], MxB [36,37,38,39,40,41], and Cyclophilin A (CypA) [33,42,43,44,45,46,47,48,49,50].

Given its key role in many diverse processes critical to replication, the HIV-1 CA protein has emerged as an attractive therapeutic target. In particular, an interprotomer pocket within the hexamer is a binding site for key host dependency factors [10,24,25] and is the target of compounds **GS-CA1** [51] and **PF-3450074** (**PF-74**) [10,24,25,52,53,54].

HIV-1 exhibits significant genetic diversity, both within and between subtypes. Different subtypes of HIV-1 can differ from 10% to 30% throughout their genomes, translating into very variable protein sequences. We have previously shown that subtype differences within the HIV-1 matrix (MA) protein have altered interaction profiles with human Calmodulin with possible biological implications [55]. This study expands the investigation of subtype differences within HIV-1, focusing on the HIV-1 CA protein. The CA protein appears to be one of the most conserved proteins of HIV-1, especially in functionally relevant regions. However, as with other HIV-1 proteins, regions outside the functionally essential areas display lower conservation. Moreover, the CA protein appears to be highly flexible, a quality that undoubtedly allows it to perform its varied functions. Given this flexibility and the variation in amino acid sequence, CA proteins from different clades likely have distinct biochemical and assembly properties. Changes around the interprotomer pocket may alter the size and shape of the pocket and therefore alter the affinity with which CA interacts with both host cellular factors and pharmacologic inhibitors. Despite the potential inter-clade differences in CA structure and function, all structural and binding studies to date have focused on the capsid protein from subtype B HIV-1 (NL4.3) [47]. Therefore, as a first step to exploring how the biochemistry and structures of the HIV-1 CA protein differ between clades, we expressed and purified monomeric and hexameric CA constructs corresponding to reference isolates from clades A1, A2, B, C, and D. We characterized their assembly properties and their interaction with host cell factors and the pharmacologic agent PF-74 using surface plasmon resonance (SPR) and compared their binding properties to the prototypical NL4-3 capsid. Additionally, we used computational methods to understand our results and to afford correlations that could be used in the design of future CA inhibitors. These new CA constructs represent novel reagents that can be used in CA-targeted inhibitor design and structural studies and will support investigations into CA function across genetically diverse HIV-1 subtypes.

## 2. Materials and Methods

### 2.1. Alignment of CA Sequences from Diverse Clades

The amino acid sequences from the capsid (CA) regions of the Gag polyproteins from HIV-1 isolates NL4-3 (47), 92UG037.1 [56], CDKTB48 [57], 92BR025.8 [58], and 94UG114.1 [59] were obtained from GenBank. Multiple sequence alignment was performed using Clustal Omega (1.2.4) (Conway Institute, Dublin, Ireland). The resulting alignment was manually adjusted using BioEdit to obtain the final figure highlighting amino acid similarities and differences. This multiple sequence alignment was used to generate a conservation plot based on the NL4-3 CA monomer structure (PDB ID 4XFX) utilizing the ConSurf Server [60]. The same sequence alignment was used to calculate the frequency by position using AnalyzeAlign from the HIV Sequence Database, Los Alamos (https://www.hiv.lanl.gov/content/sequence/ANALYZEALIGN, accessed on 15 December 2021). High-resolution figures were prepared using PyMOL. For the electrostatic complementarity (EC) calculations, Flare version 5 was used (Cresset^®^, Litlington, Cambridgeshire, UK).

### 2.2. Construction of Monomeric CA Expression Vectors from Diverse Clades

The coding sequences from the capsid (CA) regions of the Gag polyproteins from HIV-1 isolates 92UG037.1 [56], CDKTB48 [57], 92BR025.8 [58], and 94UG114.1 [59] were optimized for bacterial codon usage. Restriction enzyme sites were added for *Nde*I and *Bam*HI at the 5′ and 3′ ends of the gene, respectively, along with a 3′ sequence encoding a hexahistidine tag. These recombinant genes were then synthesized by Genscript (Piscataway, NJ, USA) and cloned into pET11a.

### 2.3. Overproduction and Purification of Monomeric CA Proteins

Overproduction of monomeric forms of the CA proteins from different clades was performed as described in Kortagere et al. [61] for the HIV-1 (NL4-3) variant. Briefly, the plasmids containing the C-terminally His-tagged CA DNA were transformed into BL21 (DE3) RIL competent cells (Agilent Technologies, Wilmington, DE, USA) and were expressed in ZYP-5052 auto-inducing media overnight at 30 °C with shaking at 225 rpm. The bacterial expressions were spun down, the supernatant discarded, and the pellets resuspended in 1x PBS pH 7.4. After the cells were lysed via sonication, the sample was subjected to ultracentrifugation, and the clarified lysate was applied to a Talon cobalt resin affinity column (Clonetech Laboratories, Mountain View, CA, USA). The bound protein was eluted from the column using 1x PBS pH 7.4 with 0.3 M imidazole, then dialyzed overnight into 20 mM Tris-HCl pH 8.0, concentrated to 120 µM, aliquoted, and stored at −80 °C.

### 2.4. In Vitro Assembly Assay

In vitro assembly of the monomeric HIV-1 CA proteins was performed using a modification of the standard CA turbidity assembly assay [62,63,64,65,66]. This assay relies upon the fact that the addition of high salt to HIV-1 CA protein causes self-assembly that can be monitored and quantified as the increase in turbidity over time, as described in Kortagere et al. [61,67]. Briefly, 75 µL of buffer was prepared, containing a final concentration of 50 mM NaH_2_PO_4_ pH 8.0 and various concentrations (1–5 M) of NaCl to assess the optimal concentration of salt needed to initiate assembly after the addition of the purified clade variants. To initiate the assembly reaction, 25 µL of 120 µM purified capsid was added to a final CA concentration of 30 µM (including matching DMSO concentrations). In the presence of compounds, the in vitro reaction contained 50 mM NaH_2_PO_4_ pH 8.0, 30 µM CA protein, 50 µM inhibitor, 3% DMSO, and 3 M NaCl. Readings were then taken at 350 nm every 30 s for 20 min to measure the increase in turbidity over time. The OD at 350 nm over the first 2 min was then plotted and fit with a linear regression to calculate the initial velocity (AU/min) of CA assembly.

### 2.5. Modeling and Generation of the HIV-1 CA Proteins from Diverse Clades

De novo models of HIV-1 CA proteins were generated using AlphaFold-2 within CoLab Notebook [68] (github.com/sokrypton/ColabFold, accessed on 10 January 2022). The models were further improved with MOLProbity [69], including the placement of hydrogens, Asn/Gln/His flips, and all-atom contacts, and the geometry was evaluated. Ramachandran plots for the models were assessed, and Ramachandran outlier residues were fixed with COOT [70]. The resulting model was further energy minimized using Flare version 5 (Cresset^®^, Litlington, Cambridgeshire, UK) with a gradient cut-off of 0.2 kcal/mol/A and 2000 iterations.

### 2.6. Overproduction and Purification of Hexameric CA Protein

Overproduction of hexameric forms of the CA proteins from different clades was obtained using plasmids containing the C-terminally His-tagged CA DNA. Efficient hexameric HIV-1 CA protein production was achieved by introducing cysteines at positions 14 and 45 and additional mutations W184A and M185A that prevent further assembly of the hexamers [71]. Briefly, plasmids were transformed into BL21 (DE3) RIL competent cells (Agilent Technologies, Wilmington, DE, USA) and were expressed in ZYP-5052 auto-inducing media overnight at 30 °C with shaking at 225 rpm. The bacterial expressions were then spun down at 4500 rpm for 45 min, the supernatant discarded, and the pellets resuspended in 1x PBS pH 7.4. After the cells were lysed via sonication, the sample was subjected to ultracentrifugation at 45,000 rpm for 45 min, and the clarified lysate was filtered through a 0.2-micrometer filter and applied to a Talon cobalt resin affinity column (Clonetech Laboratories, Mountain View, CA, USA). The bound protein was washed with 1x PBS pH 7.4 supplemented with 10 mM imidazole for 20 column volumes (CV). The bound protein was eluted from the column using 1x PBS pH 7.4 with 0.3 M imidazole and then dialyzed overnight into 20 mM Tris-HCl pH 8.0 supplemented with 200 mM β-mercaptoethanol. After the first dialysis, the sample dialysis tube was transferred into 20 mM Tris-HCl pH 8.0 and 50 mM NaCl supplemented with 20 mM β-mercaptoethanol and dialyzed for an additional 6 h. After 6 h, a final dialysis step was performed with 20 mM Tris-HCl pH 8.0 and 50 mM NaCl. Dialyzed CA protein was further purified, and hexameric CA fractions were separated using a Sepharose S200 (16/600) size-exclusion column. Hexameric CA proteins were immediately used for subsequent experiments without freeze–thaw cycles.

### 2.7. Peptides

For SPR experiments, peptides derived from host factors CPSF-6 and NUP-153 were used that represent the HIV-1 CA binding motif. The CPSF-6 peptide (313-PVLFPGQPFGQPPLG-327) and the NUP-153 peptide (1407-TNNSPSGVFTFGANSST-1423) were synthesized by GenScript Corp. (Piscataway, NJ, USA) [24,25,27,72].

### 2.8. SPR Characterization

All binding assays were performed on a ProteOn XPR36 SPR Protein Interaction Array System (Bio-Rad Laboratories, Hercules, CA, USA). The instrument temperature was set at 25 °C for all kinetic analyses. ProteOn GLH sensor chips were preconditioned with two short pulses each (10 s) of 50 mM NaOH, 100 mM HCl, and 0.5% sodium dodecyl sulfide. Then, the system was equilibrated with running buffer (1x PBS pH 7.4, 3% DMSO, and 0.005% polysorbate 20). The surface of a GLH sensor chip was activated with a 1:100 dilution of a 1:1 mixture of 1-ethyl-3-(3-dimethylaminopropyl) carbodiimide hydrochloride (0.2 M) and sulfo-N-hydroxysuccinimide (0.05 M). Immediately after chip activation, the HIV-1 CA protein constructs were prepared at a concentration of 10 μg/mL in 10 mM sodium acetate, pH 5.5, and injected across ligand flow channels for 5 min at a flow rate of 30 µL/min. Then, after unreacted protein had been washed out, excess active ester groups on the sensor surface were capped by a 5-minute injection of 1M ethanolamine HCl (pH 8.0) at a flow rate of 5 μL/min. A reference surface was similarly created by immobilizing a nonspecific protein (IgG b12 anti-HIV-1 gp120; obtained through the NIH AIDS Reagent Program, Division of AIDS, NIAID, NIH: Anti-HIV-1 gp120 Monoclonal (IgG1 b12) from Dennis Burton and Carlos Barbas) and was used as a background to correct nonspecific binding. Serial dilutions of PF-74, CPSF-6, or NUP-153 were then prepared in the running buffer and injected at a flow rate of 100 µL/min for a 50-second association phase, followed by up to a 5-minute dissociation phase using the “one-shot kinetics” capability of the ProteOn instrument [73]. Data were analyzed using the ProteOn Manager Software version 3.0 (Bio-Rad). The responses from the reference flow cell were subtracted to account for the nonspecific binding and injection artifacts. Experimental data were fitted to a simple 1:1 binding model. Experiments were performed in triplicate to obtain kinetic and equilibrium dissociation constants (K_D_).

### 2.9. Docking Calculations for PF-74

For small-molecule docking calculations, a PDB file for **PF-74** was prepared and then energy minimized using Flare version 5 (Cresset^®^, Litlington, Cambridgeshire, UK) with a root mean square (RMS) gradient cut-off of 0.2 kcal/mol/A and 10,000 iterations. The crystal structure of PF-74-bound HIV-1 capsid (PDB code: 4XFZ) and the AlphaFold-2-generated model of HIV-1 CA from clade A1 were prepared using Flare version 5 (Cresset^®^, Litlington, Cambridgeshire, UK) to allow protonation at pH 7.0 and the removal of residue gaps. A dimeric unit of the pre-prepared HIV-1 capsid protein structure was further prepared using Autodock tools [74,75,76], where essential hydrogen atoms, Kollman united atom type charges, and solvation parameters were added. The grid box for the docking search was centered around the **PF-74** binding site [74,75,76]. Docking calculations were performed using AutoDock via DockingServer [77].

### 2.10. Docking Calculations for NUP-153

For peptide docking calculations, a PDB file for NUP-153 in complex with HIV-1 CA from clade B (PDB ID 4U0C) was prepared and then energy minimized using Flare version 5 (Cresset^®^, Litlington, Cambridgeshire, UK ) with a root mean square (RMS) gradient cut-off of 0.2 kcal/mol/A and 10,000 iterations. The crystal structure of HIV-1 capsid from clade B (PDB code: 4XFX) and the AlphaFold-2-generated model of HIV-1 CA from clade A1 were prepared using Flare version 5 (Cresset^®^, Litlington, Cambridgeshire, UK) to allow protonation at pH 7.0 and the removal of residue gaps. Docking calculations were performed using Flare version 5 with a grid box set around the HIV-1 CA interprotomer pocket (**PF-74** and NUP-153 (1407-TNNSPSGVFTFGANSST-1423) binding site).

For docking calculations of the RRR patch of NUP-153 (1462–1475) into the tri-hexameric interface of CA, the fully automated FRODOCK server was used [78].

### 2.11. Interface and Interprotomer Pocket Analysis of the HIV-1 CA Hexamer

Interprotomer interfaces were assessed with PDBePISA [79], an interactive tool for the exploration of macromolecular interfaces. The volumetric and surface area of the interprotomer pocket (CTD-NTD) was computationally assessed using DoGSite (BioSolveIT, Hamburg, Germany) [80,81].

## 3. Results

### 3.1. HIV-1 CA Protein Conservation

**Figure 1:**
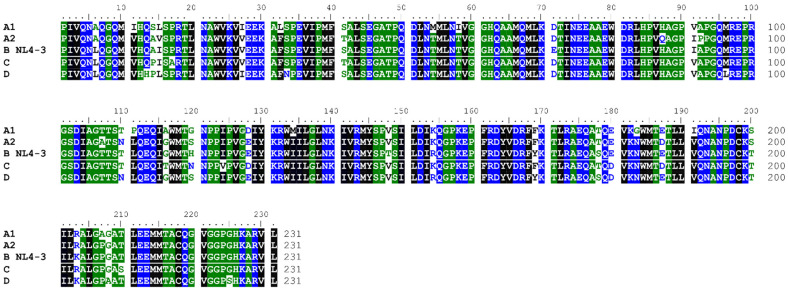
**Alignment of the amino acid sequences of the CA proteins from diverse clades.** The subtypes of the isolate are given after the isolate’s name. Identities to the CA protein from HIV-1 (NL4-3) are as follows: A1 = 89.6%; A2 = 93.1%; C = 92.6%; D = 93.1%. Blue letters indicate hydrophilic, green indicates neutral, and black indicates hydrophobic residues—shade threshold at 70%. The alignment was performed using Clustal Omega (Conway Institute, Dublin, Ireland) and manually adjusted with BioEdit.

**Figure 2:**
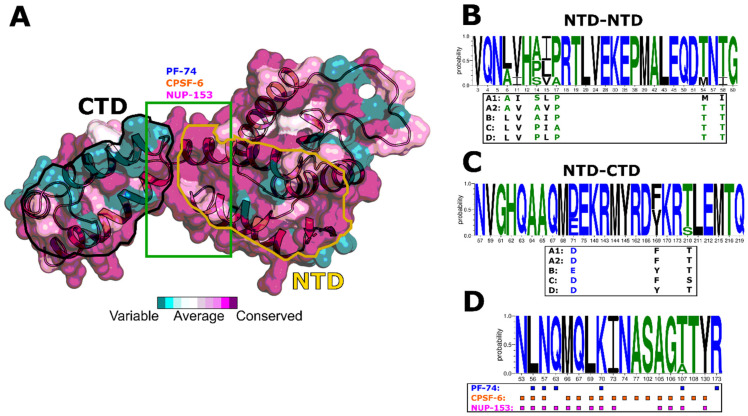
**Conservation analysis of HIV-1 CA.** (**A**) Conservation analysis of the HIV-1 CA protein based on the multiple sequence alignment (MSA) in Figure 1. Conservation is represented on the surface of HIV-1 CA from clade B (PDB code: 4XFX). The solid green box indicates the PF-74, CPSF-6, and NUP-153 binding sites; the yellow line indicates the NTD–NTD interface; and the black line indicates the CTD–NTD interface. The figure was prepared using the ConSurf Server [60]. (**B**) Conservation of major residues involved in the NTD–NTD interaction and the respective altered residues in each clade (highlighted in the box). (**C**) Conservation of major residues involved in the NTD–CTD interaction and the respective altered residues in each clade (highlighted in the box). (**D**) Conservation of major residues involved in the PF-74 (blue, PDB code: 4XFZ), CPSF-6 (orange, PDB code: 4WYM), and NUP-153 (pink, PDB code: 4U0C) interaction based on clade B. Residue numbers correspond to their placement within the Hxbc2 CA numbering. Blue letters indicate hydrophilic, green indicates neutral, and black indicates hydrophobic residues. The size of the letter indicates the probability of conservation. The sequence alignment was generated using the AnalyzeAlign tool on the Los Alamos Database (www.hiv.lanl.gov, accessed on 15 December 2021).

The HIV-1 CA protein forms a fullerene-like core structure that protects the viral genome and its essential enzymes as the capsid is translocated to the nuclear core complex (NPC). The CA protein contains multiple interface regions responsible for multimerization, which is mainly driven by NTD–NTD and NTD–CTD interactions. Moreover, the assembled capsid has multiple regions/pockets that interact with the host cell factors and facilitate both the journey to and interaction with the nucleus. Changes in these regions will have implications for host cell factor interaction and, ultimately, biology. Therefore, we performed sequence alignment to assess the inter-clade variability within CA protein sequences (Figure 1 and Figure 2). This alignment revealed that the clade A1 CA sequence differs the most from the NL4-3 CA, with 89.6% identity, followed by clade C (92.6% identity) and clades A2 and D, which have 93.1% identity (Figure 1).

We next focused on the multimerization and ligand-binding regions. Based on our conservation analysis, the NTD–NTD interface is generally well conserved between all clades (Figure 2); however, the sequences from clades A1 and A2 differ slightly from this tendency (Figure 2B). Notably, the sequence differences in these clades correspond to amino acid substitutions having similar chemical characteristics—e.g., Ala/Leu at position 6 or Ile/Val at position 11 (Figure 2B). Similarly, the NTD–CTD interface is well conserved within the clades, with the most noticeable alteration at position 169 with a Tyr to Phe for clades A1, A2, and C (Figure 2A,C).

The surface area between the NTD and CTD is highly conserved within the clades and is the target site for nuclear import factors such as CPSF-6 and NUP-153, which bind via the canonical Phenylalanine-Glycine (FG) motif that is found within the C-terminus of both nuclear import factors. A small molecule mimicking this motif, **PF-74**, also binds at this position (Figure 2A,D). Notably, a highly positively charged RRR motif located at the very C-terminus of NUP-153 was recently shown to bind to the trimeric interface of three adjacent CA hexamers, exhibiting a higher affinity than the FG motif [82]. Having identified those alterations at the sequence level prompted us to investigate whether those alterations impact host factor and small molecule recognition and, ultimately, biology.

### 3.2. Overproduction and Purification of CA Monomeric Proteins from Diverse Clades

**Figure 3:**
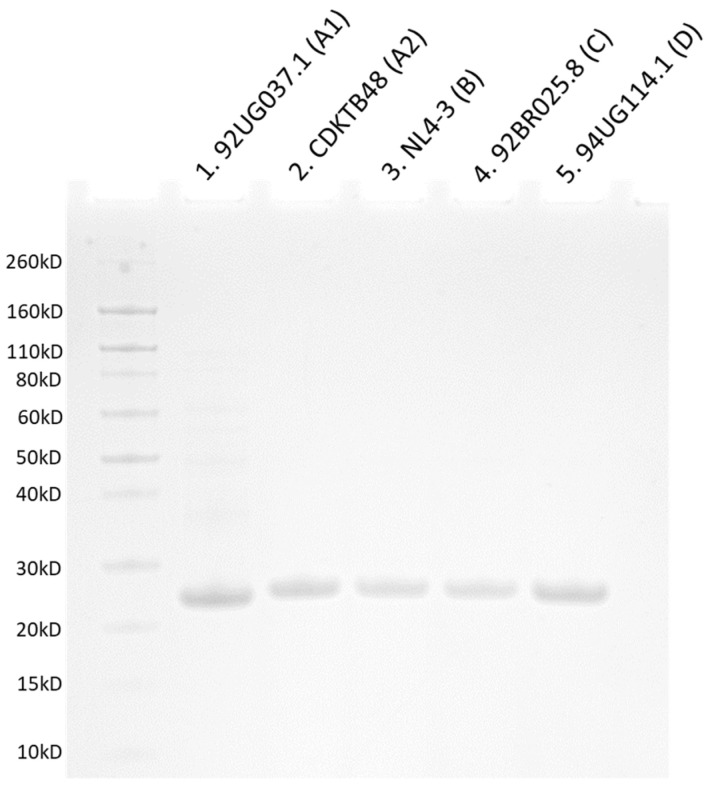
**SDS-PAGE analysis showing successful purification of CA from different clades.** Each lane shows the purified and pooled elutions from each isolate. Lane 1: 92UG037.1 (clade A1); lane 2: CDKTB48 (clade A2); lane 3: NL4-3 (clade B); lane 4: 92BR025.8 (clade C); lane 5: 94UG114.1 (clade D).

Having highlighted notable sequence alterations across the HIV-1 CA proteins from diverse clades, we were interested in whether those alterations translate into altered assembly profiles. To test this hypothesis, we overproduced and purified CA proteins from five different HIV-1 isolates: NL4-3 (clade B), 92UG037.1 (clade A1), CDKTB48 (clade A2), 92BR025.8 (clade C), and 94UG114.1 (clade D). Despite the sequence differences of these various proteins, we were able to use previously established methods to overproduce and purify all of them (Figure 3) [61,67]. However, yields varied, with the CA proteins from clades A1 and A2 giving the lowest and highest yields (95 μg/L and 820 μg/L, respectively) and intermediate yields being obtained for proteins from clades B, C, and D (360 μg/L, 214 μg/L, and 225 μg/L, respectively). Nevertheless, we were able to produce and purify enough of these new CA proteins for biochemical investigations.

### 3.3. In Vitro Assembly of CA Monomeric Proteins from Diverse Clades

**Figure 4:**
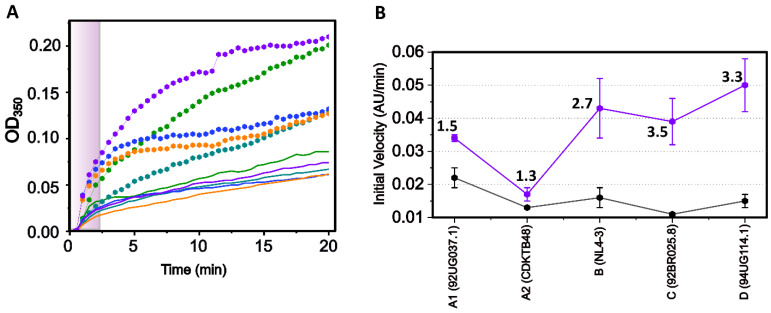
**Assembly of CA monomers from various clades in the presence of PF-74.** (**A**) Assembly of CA monomers in the presence of **PF-74** (hexagons) or vehicle control (solid lines). Clade A1 (92UG037.1) in green, clade A2 (CDKTB48) in teal, clade B (NL4-3) in blue, clade C (92BR025.8) in orange, and clade D (94UG114.1) in purple. Purple box indicates the initial 120 s. (**B**) HIV-1 CA assembly velocity increase within the initial 120 s in the presence of **PF-74**. Black data points represent Apo form, while purple represents assembly in the presence of **PF-74**. Numbers highlight the fold increase. Data points are the mean ± standard deviation with *n* = 3. Experiments were performed with 3M NaCl. *p*-value at α-level 0.05 for mean initial velocity (Apo vs. **PF-74** bound): A1 = 0.022; A2 = 0.090; B (NL4.3) = 0.039; C = 0.021; D = 0.018.

Having successfully purified CA proteins from five clades, we utilized the established CA assembly assay to characterize the assembly profile and ensure that the recombinant proteins functioned correctly [66,83,84]. In brief, the assembly of CA into tubes is induced upon the addition of high concentrations of NaCl. During the assembly process, the turbidity of the reaction increases. Therefore, measuring the increase in absorbance at 350 nm over time due to the increase in turbidity directly measures the assembly of the CA tubes. First, we performed the in vitro assembly assay on the purified CA from each clade in the presence of different salt concentrations to determine the optimum concentration to use for each clade. Each clade displayed slightly different assembly profiles in the presence of varying salt concentrations (1–5 M NaCl; data not shown). Although the maximum absorbance varied slightly across the clades, it was still clear that all clades assembled best in the presence of 3M NaCl. Therefore, we compared the assembly of each clade in the presence of 3M NaCl to observe the differences in assembly efficiency and compare across clades. The overlay of each clade along with the linear regression fits to determine the initial velocity (first 120 s) revealed some slight differences in the assembly rates between each clade (Figure 4 solid lines and Table 1). Clade A1 assembled the fastest, followed by clades B and D. HIV-1 CA from clades C and A2 assembled at the slowest velocity (Table 1).

Several small-molecule compounds have been shown to either disrupt or increase the assembly of CA in vitro [67,83,84,85,86]. Since it has been shown that **PF-74** promotes the assembly of NL4-3 CA protein from clade B [52,85,87], we wanted to see if this inhibitor elicited the same effects on the assembly of CA proteins from other clades. As expected, adding **PF-74** to the CA monomer assembly assay increased the assembly rates but to various degrees for each clade (Figure 4 dotted lines). Proteins from clades A1 and A2 displayed the smallest increase in assembly rate upon **PF-74** binding (1.5- and 1.3-fold increase, respectively), while proteins from the other clades all showed a roughly three-fold increase in assembly rate (Figure 4, Table 1). Investigation of the A1 and A2 sequences revealed that they contain changes relative to the NL4-3 sequence found in the “5mut” CA, which confers **PF-74** resistance [88]. The five mutations that constitute the 5mut mutant are all located in the N-terminus, away from the actual binding site of **PF-74**. Interestingly, the capsid cores isolated from the 5mut HIV-1 show increased stability resulting from the mutations in the NTD–NTD interface [89]. This indicates that a change within the NTD may alter the assembly interface, thereby affecting the rate and stability of assembly and also having structural consequences for the interprotomer binding pocket that is the binding site of **PF-74** (and host cell factors). The initial velocities of the clade A1 and A2 capsid proteins are the fastest and slowest, respectively. They are also the least responsive to PF-74-induced increases in assembly, indicating that the assembly rates may be a controlling factor for **PF-74** resistance. Taken together, we showed that the slight sequence alterations across clades do translate into altered assembly rates of the Apo protein and an even more dramatically altered assembly increase upon **PF-74** binding. The effect of **PF-74** binding on the assembly of each CA clade indicates that the interprotomer pocket is still formed to some degree within each of the clades despite the sequence differences. Therefore, to obtain a more comprehensive understanding of the alterations within the interprotomer pocket, we next wanted to explore stabilized CA hexamers from each clade.

### 3.4. Overproduction and Purification of CA Hexameric Proteins from Diverse Clades

**Figure 5:**
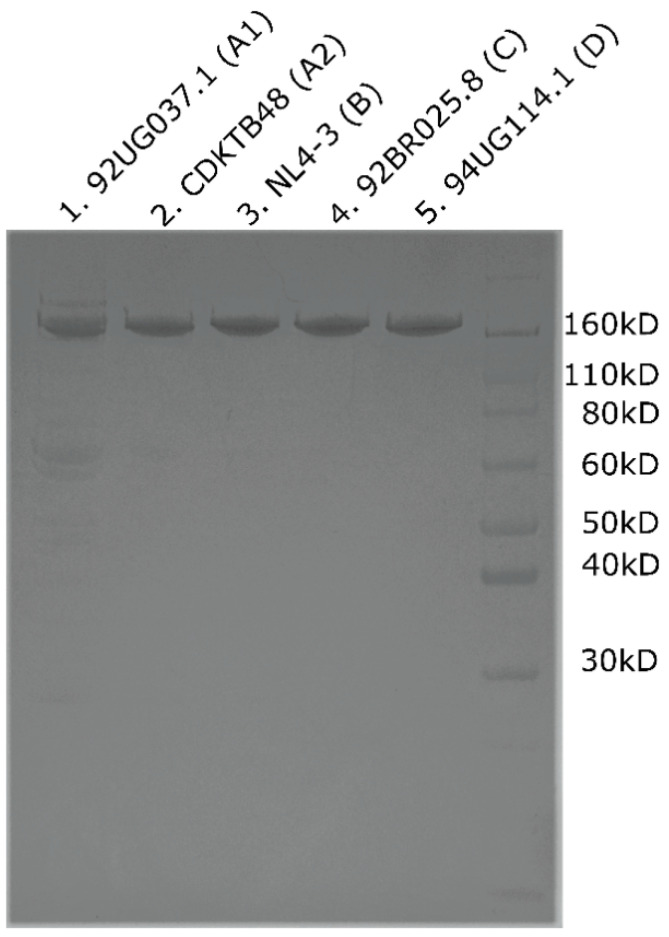
**Non-reducing SDS-PAGE analysis shows successful purification and hexamerization of CA from different clades.** Each lane shows the purified and pooled elutions from each isolate. Lane 1: 92UG037.1 (clade A1); lane 2: CDKTB48 (clade A2); lane 3: NL4-3 (clade B); lane 4: 92BR025.8 (clade C); lane 5: 94UG114.1 (clade D).

As mentioned in the previous section, assembly of the CA protein into hexamers is critical for creating the fullerene cone and creating binding pockets for host cell factors and pharmacologic compounds. Therefore, in order to gain more insight into clade-specific differences, we needed to convert the non-clade-B monomers into hexamers. A stable hexameric version of the prototypical HIV-1 NL4-3 CA protein was engineered by Pornillos et al. [8]. Therefore, we engineered the new clade constructs with the same amino acid substitutions to convert them into stable hexamers too. We introduced cysteine residues (at positions 14 and 45) that promote hexamer formation by forming interprotomer disulfide bonds and mutations W184A and M185A that prevent further assembly of the hexamers [71]. These proteins were purified in the presence of 200 mM β-mercaptoethanol, after which hexamer formation was initiated by removing the reducing agent via dialysis. Similar to the monomeric CA protein expression, slight differences in yields and hexamerization efficiency were observed within the clades. Protein from clade A1 had the lowest yield of 360 μg/L, clade A2 had a yield of 500 μg/L, NL4-3 (clade B) had a yield of 3800 μg/L, clade C had a yield of 480 μg/L, and clade D had a yield of 1800 μg/L. Clade A1 also displayed the lowest hexamerization yield compared to the other clades. Nevertheless, each of the five constructs was successfully purified and hexamerized, as can be seen by SDS-PAGE analysis, with bands for each hexameric isolate appearing at around their predicted size, just above 160 kDa (Figure 5).

### 3.5. Altered Substrate Recognition across Hexameric CA Proteins from Diverse Clades

Given our observation that **PF-74** affects the different monomeric CA proteins differently, coupled with the sequence changes within the interprotomer pocket and NTD–NTD interface (see Figure 2) between the clades, we sought to see whether this translated to differences in the interaction with **PF-74** and host cell proteins. Therefore, in addition to **PF-74**, we examined whether the different hexameric CA proteins bound differently to peptides derived from two host cell factors essential for the nuclear import of the HIV-1 CA core: CPSF-6 and NUP-153. These two import factors facilitate import in distinct ways: while NUP-153 stabilizes the CA core structure during NPC translocation, CPSF-6 destabilizes and therefore defines the spatial presence of the pre-integration complex, vital for viral genome integration into the host genome.

**Figure 6:**
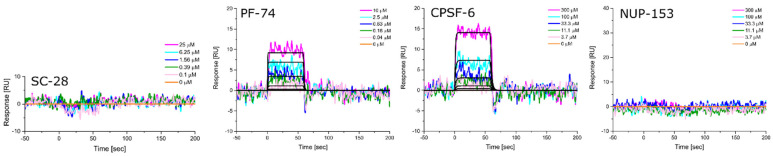
**Representative sensorgrams for HIV-1 CA hexamer from clade A1 to SC-28 (an entry Env inhibitor), PF-74, CPSF-6, and NUP-153.** Colored lines represent data collected from the dilution series, whereas black lines signify the fits to a 1:1 binding model. Interaction parameters derived from a triplicate (*n* = 3) of data given in Table 2, Table 3 and Table 4.

**Figure 7:**
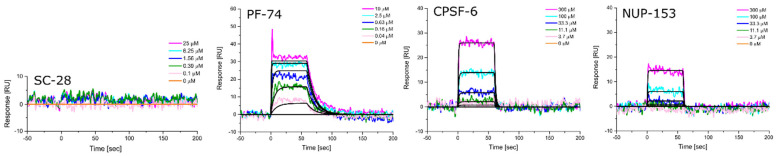
**Representative sensorgrams for HIV-1 CA hexamer from clade A2 to SC-28 (an entry Env inhibitor), PF-74, CPSF-6, and NUP-153.** Colored lines represent data collected from the dilution series, whereas black lines signify the fits to a 1:1 binding model. Interaction parameters derived from a triplicate (*n* = 3) of data given in Table 2, Table 3 and Table 4.

**Figure 8:**
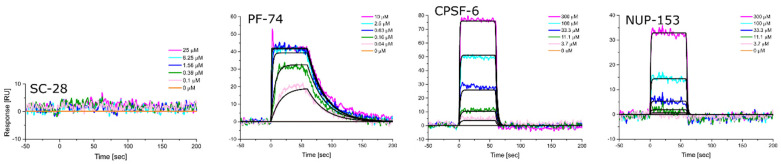
**Representative sensorgrams for HIV-1 CA hexamer from clade B to SC-28 (an entry Env inhibitor), PF-74, CPSF-6, and NUP-153.** Colored lines represent data collected from the dilution series, whereas black lines signify the fits to a 1:1 binding model. Interaction parameters derived from a triplicate (*n* = 3) of data given in Table 2, Table 3 and Table 4.

**Figure 9:**
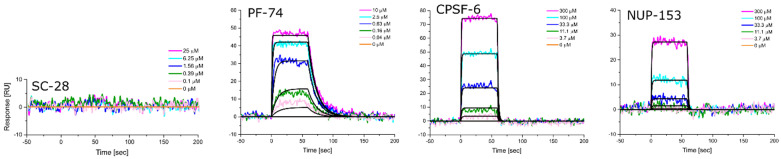
**Representative sensorgrams for HIV-1 CA hexamer from clade C to SC-28 (an entry Env inhibitor), PF-74, CPSF-6, and NUP-153.** Colored lines represent data collected from the dilution series, whereas black lines signify the fits to a 1:1 binding model. Interaction parameters derived from a triplicate (*n* = 3) of data given in Table 2, Table 3 and Table 4.

**Figure 10:**
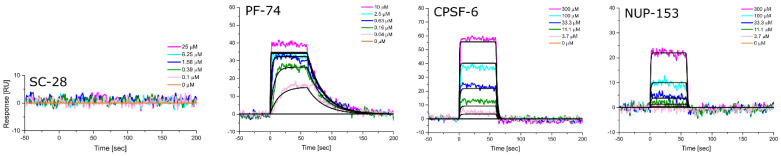
**Representative sensorgrams for HIV-1 CA hexamer from clade D to SC-28 (an entry Env inhibitor), PF-74, CPSF-6, and NUP-153.** Colored lines represent data collected from the dilution series, whereas black lines signify the fits to a 1:1 binding model. Interaction parameters derived from a triplicate (*n* = 3) of data given in Table 2, Table 3 and Table 4.

**Figure 11:**
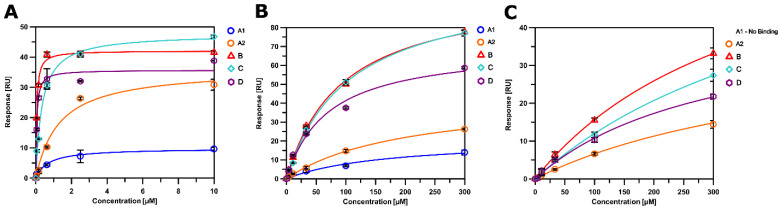
**Binding isotherms of PF-74 (A), CPSF-6 (B), and NUP-153 (C) to immobilized HIV-1 CA hexamer from clades A1, A2, B, C, and D.** Binding isotherms are derived from the data in Figure 7, Figure 8, Figure 9, Figure 10 and Figure 11. Experiments were performed in triplicate, and data are displayed as the mean ± standard deviations (SD).

Equipped with hexameric HIV-1 CA proteins from diverse clades, we used surface plasmon resonance (SPR) spectrometry to determine the equilibrium dissociation constants (K_D_, at equilibrium, and from global kinetics) and kinetic profiles for **PF-74**, CPSF-6, and NUP-153 binding. We first used **SC28**, a potent HIV-1 inhibitor targeting the Env machinery, as a negative control [90,91,92]. As expected, **SC28** did not bind to any of the CA hexamers studied (Figure 6, Figure 7, Figure 8, Figure 9 and Figure 10, left panel). **PF-74** bound, as expected, to proteins from all clades but with different affinities. While proteins from clades B (NL4-3) and D recognized **PF-74** with low nanomolar affinities, the protein from clade A2 showed an almost three-fold lower affinity (Figure 7, Figure 8, Figure 10, Figure 11A and Table 2), and the affinities for the proteins from clades C and A1 fell into the high nanomolar range (Figure 8, Figure 9, Figure 11A and Table 2). Interestingly, binding to the clade C protein displayed a slower on-rate but an identical off-rate compared to clade B, explaining the lower affinity overall. Reciprocally, the clade A1 protein bound with a similar on-rate to **PF-74** compared to clade B but with a faster off-rate, which also explains the lower overall affinity (Table 2).

We next determined the binding profile of CPSF-6 to all clades. We identified that the clade D protein had the lowest affinity accompanied by the fastest on-rate (Figure 10, Figure 11B and Table 3), followed by clades B, C, A1, and A2 (Figure 8, Figure 9, Figure 11B and Table 3) (Figure 6, Figure 7 and Figure 11B). Close investigation revealed that CA from clade A1 bound to CPSF-6 with a slower on- and off-rate as compared to that from A2, balancing the overall affinity in the same order of magnitude between A1 and A2 (Table 3).

Clade B and clade D proteins showed the highest affinities for binding to NUP-153, displaying comparable kinetic profiles (Figure 8, Figure 9, Figure 11C and Table 3); proteins from clades A2 and C showed slightly lower affinities, with the clade C protein also displaying the slowest on-rate out of all the clades (Figure 7, Figure 9, Figure 11C and Table 4). Interestingly, we could not detect any binding of clade A1 CA to NUP-153, possibly reflecting the lowest sequence identity between this protein and the clade B protein (Figure 6, Figure 11C and Table 4). Thus, while the CA proteins from the different clades bind similarly to CPSF-6, they differ significantly in their recognition of **PF-74** and even more dramatically so in their recognition of NUP-153. Hence, consistent with our findings for monomeric CA proteins, sequence variations between clades also affect hexameric CA proteins and their substrate recognition. To our knowledge, this work is the first to demonstrate that CA proteins from diverse clades differ in their assembly rates and recognition of binding partners. This study also highlights the value of quantifying the dynamic kinetics of interactions in addition to overall affinity.

### 3.6. Modeling of Non-Subtype-B CA Proteins—Implications for Assembly

**Figure 12:**
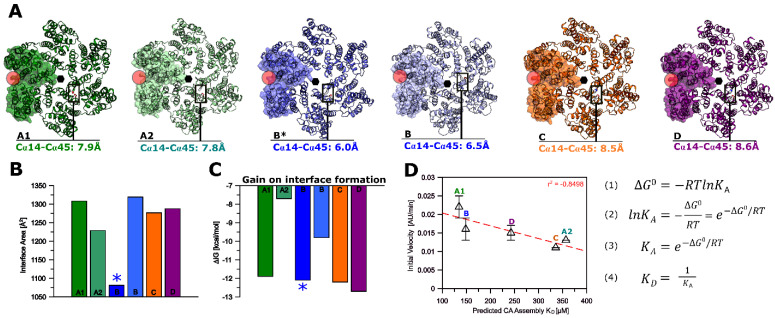
**AlphaFold-2 models and interface analysis of HIV-1 CA hexamers of clades A1, A2, B, C, and D.** (**A**) AlphaFold-2 models of HIV-1 CA hexameric assemblies. Asterisks indicate the crystal structure of HIV-1 CA from clade B (NL4-3). Hexamers were structurally aligned to the clade B (PDB ID 4XFX) crystal structure. The red circle indicates the binding site for **PF-74**, CPSF-6 (313–327), and NUP-153 (1407–1423). The black box highlights the location of Cys14 and Cys45 Cα of two neighboring protomers with the respective distance in Å. A1 vs. B (PDB ID 4XFX) r.m.s.d.: 1.605 Å (over 1272 Cα atoms); A2 vs. B (PDB ID 4XFX) r.m.s.d.: 1.345 Å (over 1293 Cα atoms); B (AlphaFold-2) vs. B (PDB ID 4XFX) r.m.s.d.: 0.794 Å (over 1266 Cα atoms); C vs. B (PDB ID 4XFX) r.m.s.d.: 1.292 Å (over 1284 Cα atoms); D vs. B (PDB ID 4XFX) r.m.s.d.: 1.076 Å (over 1290 Cα atoms). (**B**) Interface area in Å between two adjacent protomers. (**C**) Gain on the complex formation (Δ^i^G) in kcal/mol. (**D**) Initial velocity in AU/min vs. predicted K_D_ according to Equations (1)–(4). R = 0.001987 kcalmol^−1^K^−1^, K = 310.15 K. Interface analysis was performed using the PDBePISA server.

We have, thus far, demonstrated experimentally that CA proteins from diverse clades differ in assembly rates and recognition of binding partners. To understand the possible molecular principles behind our findings, we generated de novo models of the CA proteins from the various clades using AlphaFold-2 (Figure 12A).

Not surprisingly, the proteins from all the clades display a very similar overall hexameric arrangement with Cα root mean square deviations (r.m.s.d.) ranging from 1.076 Å (clade D) to 1.605 Å (clade A1), using clade B as a reference (PDB ID 4XFX). As the engineered Cys14 and Cys45 residues are critical for yield and stability during hexamer preparation, we additionally measured the Cα distance of Cys14 to Cys45 in all clades and identified clade B with the shortest distance of 6.0 Å as compared to the other clades, a finding in line with clade B yielding the highest amount of hexamer formation during preparation. Next, we investigated the interface between protomers of all clades using the PDBePISA server and found that the interfaces between two protomers are similar across all clades except for A2, which shows the smallest interface surface area (Figure 12B). Based on this analysis, the solvation free energy gain (Δ^i^G) upon the formation of the interface suggests the most favorable gain for clade D, followed by clades C, B, A1, and A2 (Figure 12C). Note that this free energy gain (Δ^i^G) does not include hydrogen bonds and salt bridge formation. We therefore used the average solvation energy gain (ΔG^ave^) to derive an approximate K_D_ for complex formation. The predicted K_D_ values correlate well (r^2^ = |0.8498|) with each clade’s experimentally derived assembly rates and thus appear to be useful indicators of assembly efficiency (Figure 12D).

**Figure 13:**
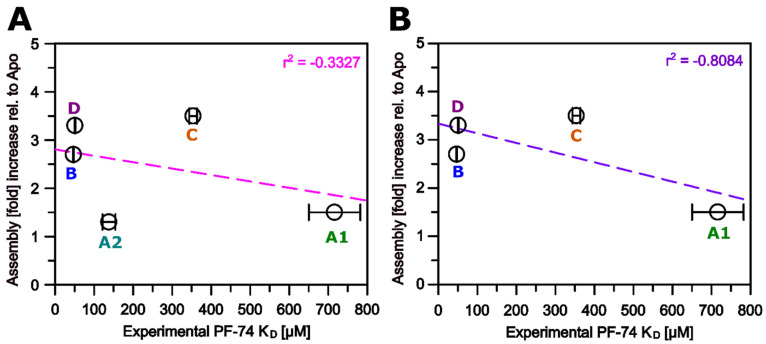
**Assembly increase in the presence of PF-74 vs. experimentally derived PF-74 K_D_.** (**A**) Fold increase in initial velocity in AU/min upon **PF-74** binding for all clades. (**B**) Fold increase in initial velocity in AU/min upon **PF-74** binding, excluding HIV-1 CA from clade A2.

We next tested whether the binding affinity for **PF-74** correlates with the increase in assembly rates elicited by this compound. Using the experimentally derived affinities for **PF-74** (SPR), we noticed a weak correlation with assembly increase (r^2^ = |0.3327|), in which the clade A2 protein is an outlier (Figure 13A). This might reflect that the clade A2 protein, despite having a high affinity for **PF-74** (K_D_ of 138 nM), has a low assembly rate and the lowest predicted affinity for hexamerization, which PF-74 cannot overcome. Excluding this protein from the regression analysis increased the correlation significantly for the proteins from the remaining clades (r^2^ = |0.8084|, Figure 13B). Hence, the increase in assembly rate elicited by **PF-74** appears to be moderately linked to the drug’s affinity for clades other than A2; for the A2 protein, assembly might be hampered by its smaller interface area and lower energy gain upon hexamerization, limiting the ability of PF-74 to drive hexamerization.

### 3.7. Modeling of Non-Subtype-B CA Proteins—Implications for PF-74 and NUP-153 Binding

Next, we tried to shed light on the molecular mechanism of **PF-74** and NUP-153 recognition using our de novo generated models. We focused on understanding the interaction of **PF-74** and NUP-153 with capsomers from clades B and A1. This is because these two clades showed significant differences relative to each other in our SPR experiments, with clade B interacting robustly with both ligands and A1 only interacting with **PF-74**.

**Figure 14:**
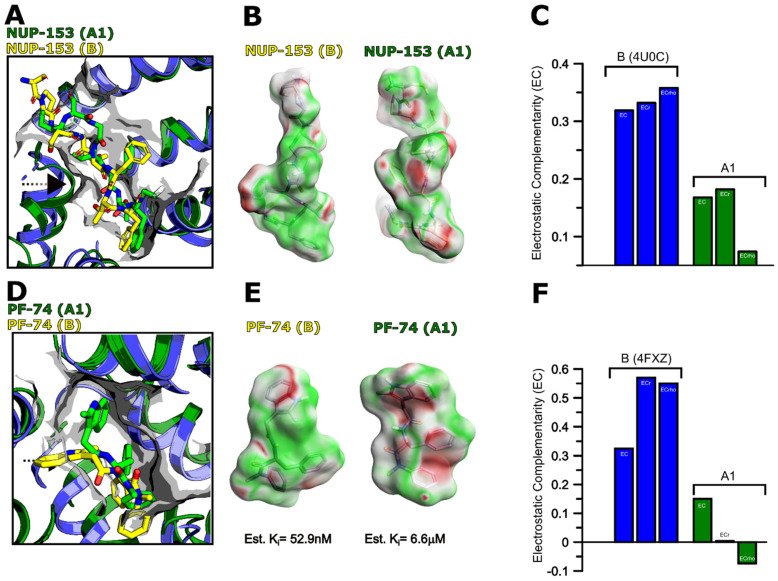
**NUP-153 and PF-74 recognition by HIV-1 CA from clades B vs. A1.** (**A**,**D**) Structural alignment of NUP-153/**PF-74** (in yellow) bound to HIV-1 CA from clade B (in blue, PDB IDs 4U0C and 4XFZ, respectively) vs. docked NUP-153 (in green) to CA from clade A1 (in green). NUP-153 (1407–1423) was docked to CA from clade A1 using Flare version 5 (Cresset^®^, Litlington, Cambridgeshire, UK). (**B**,**E**) Electrostatic complementarity (EC) comparison of docked NUP-153/PF-74 to clade A1 vs. experimentally derived clade B structures. **PF-74** was docked into the interprotomer pocket using Autodock, and an estimated inhibitory constant (Ki) is shown for each clade. (**C**,**F**) EC scores for depiction in B and E. EC: Normalized surface integral of the complementarity score; ECr: Pearson correlation coefficient; ECrho: Spearman rank correlation coefficient.

**Figure 15:**
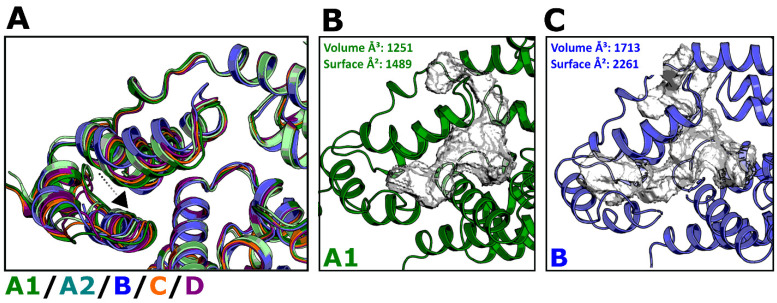
**Interprotomer pocket (CTD–NTD binding site) from clade B vs. A1.** (**A**) Overlay of all HIV-1 CA clades studied here. The CTD of clade A1 relative to B is shifted towards the NTD, as indicated by the black arrow. (**B**,**C**) Volumetric and surface assessment of the binding site between CTD and NTD from clade B vs. A1. Computational assessment of the volumetric and surface area of the interprotomer pocket was performed using DoGSite from BioSolveIT (Hamburg, Germany) [80,81].

NUP-153 (1407–1423) and **PF-74** bind within the NTD–CTD interprotomer pocket (Figure 2), and the crystal structure of HIV-1 CA from clade B in complex with NUP-153 indicates that the peptide binds in an elongated conformation between the two domains. This extended conformation allows the insertion of the Phenylalanine-Glycine (FG) motif into a hydrophobic pocket that is formed between two protomers (Figure 14A, yellow from PDB ID 4U0C). We docked the NUP-153 peptide into this pocket using the clade A1 AlphaFold-2 model and compared the complex with CA’s NUP-153-bound crystal structure from clade B. Since the residues involved in NUP-153 recognition are highly conserved (Figure 2), we first chose to look at the electrostatic complementarity (EC) between a ligand and receptor. The EC of a ligand–receptor complex can provide essential insights into the nature and strength of the complex [55]. Since electrostatic interactions are key contributors to the enthalpic component of the free energy of binding, we chose to look at the EC map of NUP-153 bound to proteins from clades B and A1 and compare EC values with our experimentally derived K_D_ values. The structural overlay of both complex structures indicates that the CTD of the AlphaFold-2 model of clade A1 is slightly shifted towards the NTD (Figure 14A and Figure 15A, indicated by the black arrow). This shift could potentially reduce the size and volume of the interprotomer binding site in clade A1, resulting in NUP-153 having to adopt a more compact conformation than is indicated by the crystal structure of clade B (compare in Figure 14A, yellow vs. green NUP-153). The net result of this could be drastically unfavorable binding conditions for NUP-153 to A1 as a result of an insurmountable entropic penalty of binding. Indeed, if we quantify the surface (Å^2^) and volume (Å^3^) within this interprotomer area, we see a reduction in both the surface of 2261 Å^2^ and in the volume of 1713 Å^3^ from clade B as compared to clade A1 with a surface of 1489 Å^2^ and a volume of 1251 Å^3^ (Figure 15B,C), suggesting that the lack of binding could be a result of the change in dimension of the interprotomer pocket.

As the electrostatic complementarity (EC) of a ligand with its binding pocket influences the affinity of the interaction, especially small peptides, we chose, again, to quantify the EC. Based on this analysis, in addition to the smaller interprotomer pocket of clade A1, differences in the EC map suggest an apparent reduction in electrostatic complementarity for NUP-153 binding to clade A1. This is highlighted in Figure 14B,C, with the green surface area representing good complementarity and the red surface area representing an electrostatic clash (and the white surface suggesting neutral electrostatics). We believe that EC reduction and the smaller interprotomer pocket provide a plausible explanation for the complete loss of binding of NUP-153 to clade A1. Interestingly, the peptide from CPSF-6 did bind with comparable affinities across all HIV-1 clades within this study (Table 2), including clade A1. The conformation and space within the interprotomer pocket occupied by the CPSF-6 peptide are more compact and smaller than the NUP-153 conformation. As such, we believe that whilst NUP-153′s extended binding mode is incompatible with the reduced interprotomer pocket in the A1 hexamer, CPSF-6’s smaller bioactive conformation is compatible [24,72].

Similarly, we looked at **PF-74** binding to proteins from clades B and A1. Due to its smaller size and volume, **PF-74** is unlikely to be as affected by the predicted reduction in interprotomer pocket area as the extended NUP-153 peptide is. However, our SPR data show that **PF-74** binds ~20-fold more tightly to the clade B protein as compared to the clade A1 protein, highlighting an appreciable difference in interaction. When comparing the crystal structure of **PF-74** bound to the clade B protein against our docked A1 complex (Figure 14D), we noticed that there is a slight difference in the binding modes of **PF-74** in the pocket of the clade B capsid and our clade A1 capsid model. This minor difference could affect the affinity in two ways—introducing an entropic penalty and clash and throwing off the electrostatic complementarity between PF-74 and the pocket. Upon closer inspection of the bioactive conformation of PF-74 bound to the clade A1 capsomer, it is apparent that the 2-methyl-indole moiety of **PF-74** in A1 is kinked, and this small change in conformation does throw off the EC and introduce the potential for clashes, etc. (Figure 14E,F). This predicted altered **PF-74** binding mode and its resultant unfavorable binding consequences for its interaction with the clade A1 capsomer provide a plausible explanation for the observed differences in affinity and kinetics of interaction for clade A1 [87].

In summary, docking calculations with the de novo derived CA model from clade A1 combined with the experimentally obtained clade B structures were used to shed light on the underlying molecular mechanism we observed in our SPR study. The computationally derived values correlate well with the experimentally derived K_D_ values.

**Figure 16:**
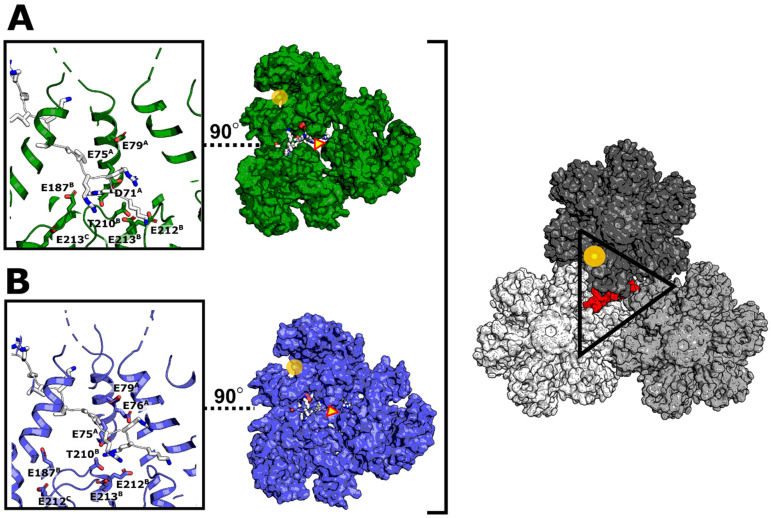
**Tri-interhexamer binding site (“RRR” patch) for C-terminal portion of NUP-153 (1462–1475—“RRR” patch).** (**A**) Docked C-terminal portion of NUP-153 (1462–1475) to the tri-hexameric interface of HIV-1 CA clade A1. (**B**) Docked C-terminal portion of NUP-153 (1462–1475) to the tri-hexameric interface of HIV-1 CA clade B (PDB ID 6ECO). The yellow circle highlights the FG patch binding site (interprotomer pocket or CTD–NTD). The triangle highlights the tri-interhexameric interface. NUP-153 (1462–1475) was docked into the tri-hexameric interface using FRODOCK [78] and energy minimized using Flare version 5.

We showed experimentally and supported computationally that NUP-153 (1407–1423) does not bind to HIV-1 CA from clade A1. However, the C-terminal portion of NUP-153 binds to CA with two consecutive patches—the Phenylalanine-Glycine (FG) patch (analyzed in this study), which binds with low affinity, and a recently discovered RRR patch that binds with higher affinity (K_D_ of 800 nM and stoichiometry of 1.0)—into the tri-hexameric interface comprised of mainly negatively charged Glu, Asp, and also Thr residues [82]. HIV-1 CA from clade A1 does have a very similar negatively charged tri-hexameric interface (Figure 16A), and we hypothesized that the RRR patch (within the tri-hexameric interface) could compensate for the lack of binding to the Phenylalanine-Glycine (FG) patch by NUP-153. Rigid docking of the NUP-153 (1462–1475) RRR patch into this interface resulted in very similar orientations of the peptide for both the clade A1 and clade B proteins (Figure 16A,B). Further experimental evaluation of this observation is necessary to confirm the binding of the RRR patch to the tri-hexameric interface of the clade A1 protein, which could compensate for the lack of Phenylalanine-Glycine (FG) patch binding to the interprotomer pocket.

## 4. Discussion

Due to its critical involvement in the HIV-1 lifecycle, the CA protein has become a target of inhibition and structural studies. Most studies on the CA protein and its numerous multimeric states (monomeric, hexameric, pentameric, and the fullerene core) have been performed on the NL4-3 isolate from clade B. However, the CA protein has been shown to be genetically fragile, indicating that sequence alterations within the CA protein between clades can have an impact on biology and therapeutic inhibition. During the assembly of a budding virion, CA multimerization is an essential step for the generation of an infectious virion. After infection occurs, the stability of the CA core is critical for numerous steps within the early stages of the HIV-1 lifecycle, such as reverse transcription, transport, and nuclear import. CA hexamers provide multiple contact points for interaction with host import factors during translocation of the HIV-1 core structure with the viral genome through the NPC. Those factors include NUPs (62, 88, 214, 358, and 153), with NUP-153 being the most critical; this protein is located at the pore’s nucleus side and prefers to bind to high curvature regions of the CA cone.

Sequence variation in viral proteins is significant for both biology and therapies. When comparing the sequences of CA proteins from various clades, we observed a significant difference in the amino acid sequence, where the different residues were mainly present in the protomer interface. As HIV-1 CA assembly is critical for its biological function, and this interface is also the target of numerous therapeutic strategies, we believe that extensive investigation of this interface will have implications for future drug design efforts. The de novo models used in this study could support early detection of natural resistance to antivirals, improve antiviral design, adjust the therapeutic strategy accordingly, and open a new way to design next-generation therapies.

This gave us the rationale to overproduce, purify, and determine how this change in sequence alters the assembly function of the CA proteins. After determining the assembly rates of the purified monomeric versions of the CA proteins from five different clades, it was clear that the proteins from each clade assemble differently with varying assembly rates. These differences provide strong motivation for future structural and interaction studies to understand the biology of the non-subtype-B variants of hexameric CA.

Furthermore, we characterized the affinity and kinetic profiles of **PF-74**, CPSF-6, and NUP-153 binding to CA proteins from different clades and discovered nuanced but also drastic differences between clades. Notably, and perhaps crucial for future therapeutic interventions of CA assembly across clades, we noticed that HIV-1 CA from clade A2, despite binding **PF-74** with high affinity, did not show a drastic increase in assembly rate, which is most likely a function of the small interface area between protomers in the hexameric context. Similarly, the clade A1 protein displayed a small increase in assembly rate upon **PF-74** binding, most likely due to decreased **PF-74** affinity. These observations highlight the importance of using of crystal structures (or, in our case, models) and looking outside of the simple binding site alongside biophysical measurements to understand the mechanism of action of compounds for therapeutic targets.

Furthermore, and excitingly, we observed a complete loss of binding of NUP-153 (1407–1423) by HIV-1 CA from clade A1 (within the FG patch), a finding that our computational prediction supports. This highlights the importance of such de novo models for biological investigation and hypothesis evaluation.

During nuclear translocation of the HIV-1 CA cone, CPSF-6 competes with NUP-153 for the FG patch (interprotomer pocket) in order to destabilize the cone and initiate the release of the genome for the formation of the pre-integration complex (PIC). This process is balanced, and our findings suggest that HIV-1 CA from clade A1 might bind NUP-153 with a different mechanism as compared to the other clades studied here, thus shifting the spatially balanced CPSF-6–NUP-153 competition and possibly altering PIC formation for isolates from this clade. The lack of binding for HIV-1 CA from clade A1 could indicate that NUP-153 binds the tri-hexameric interface of CA hexamers within the cone structure to compensate for the FG patch site, or that other NUPs compensate for NUP-153′s failure to bind; both assumptions need experimental validation, which exceeds the scope of this present study, to further elucidate clade-specific differences in nuclear translocation.

## 5. Conclusions

We overproduced and purified HIV-1 CA proteins from a diverse set of clades (A1, A2, B, C, and D) for the first time, characterized their biochemical assembly properties, and demonstrated different assembly rates between the HIV-1 clades. Our in silico generated models of HIV-1 CA proteins from different clades provided structural information regarding the assembly interface, and the interface analysis correlated well with our experimentally determined assembly rates, demonstrating the feasibility of using such de novo models for biological deductions and in combination with electrostatic complementarity (EC) analysis for future antiviral design.

We demonstrated that CA hexamers from different clades have different interaction properties with host cell factors and showed a complete loss of binding of clade A1 CA to a NUP-153 peptide. Furthermore, we found a weak correlation between **PF-74** affinity and assembly increase of HIV-1 CA. The docking and electrostatic complementarity analysis provided additional molecular explanations for those alterations.

## Figures and Tables

**Table 1:** **The initial velocity (first 120 s) of HIV-1 CA assembly from each clade—Apo vs.** **PF-74**.

HIV-1 Clade	Initial Velocity Apo (AU/min)	r^2^	Initial Velocity PF-74 (AU/min)	r^2^
A1 (92UG037.1)	0.022 ± 0.003	0.966	0.034 ± 0.001	0.996
A2 (CDKTB48)	0.013 ± 0.000	0.990	0.017 ± 0.002	0.983
B (NL4-3)	0.016 ± 0.003	0.945	0.043 ± 0.009	0.925
C (92BR025.8)	0.011 ± 0.000	0.929	0.039 ± 0.007	0.930
D (94UG114.1)	0.015 ± 0.002	0.954	0.050 ± 0.008	0.955

r^2^ = Coefficient of determination.

**Table 2:** **PF-74 kinetic and equilibrium parameters for HIV-1 CA from clades A1, A2, B, C, and D.** Equilibrium dissociation constant (K_D_) derived from a Langmuir isotherm equilibrium fit or from a global fit and the derived kinetic data.

HIV-1 Clade	K_D_ [nM] Equilibrium	±SD	k_on_ [M^−1^s^−1^]	±SD	k_off_ [s^−1^]	±SD	K_D_ [nM] Kinetic/Global	±SD
A1	716.3	66.1	6.15 × 10^5^	1.80 × 10^5^	6.47 × 10^−1^	2.82 × 10^−1^	946.5	313.5
A2	138.0	16.5	6.69 × 10^5^	1.78 × 10^5^	9.16 × 10^−2^	1.42 × 10^−2^	139.3	14.6
B (NL4.3)	47.0	0.6	7.56 × 10^5^	4.55 × 10^4^	3.63 × 10^−2^	6.92 × 10^−3^	47.9	1.2
C	353.7	10.0	2.28 × 10^5^	2.20 × 10^4^	7.54 × 10^−2^	6.92 × 10^−3^	330.0	14.7
D	51.0	1.3	7.73 × 10^5^	4.01 × 10^4^	3.95 × 10^−2^	5.25 × 10^−3^	51.0	4.1

**Table 3:** **CPSF-6 kinetic and equilibrium parameters for HIV-1 CA from clades A1, A2, B, C, and D.** Equilibrium dissociation constant (K_D_) derived from a Langmuir isotherm equilibrium fit or from a global fit and the derived kinetic data.

HIV-1 Clade	K_D_ [μM] Equilibrium	±SD	k_on_ [M^−1^s^−1^]	±SD	k_off_ [s^−1^]	±SD	K_D_ [μM] Kinetic/Global	±SD
A1	181.0	55.9	1.85 × 10^3^	9.29 × 10^1^	4.77 × 10^−1^	3.27 × 10^−2^	257.3	7.8
A2	197.0	35.4	3.68 × 10^3^	1.33 × 10^3^	7.46 × 10^−1^	1.73 × 10^−1^	209.7	28.2
B (NL4.3)	93.8	3.3	5.96 × 10^3^	1.28 × 10^3^	5.89 × 10^−1^	1.28 × 10^−1^	98.6	4.7
C	104.3	3.8	5.29 × 10^3^	1.21 × 10^3^	5.70 × 10^−1^	1.28 × 10^−1^	107.7	1.5
D	70.0	0.7	7.06 × 10^3^	1.14 × 10^3^	5.13 × 10^−1^	8.05 × 10^−2^	72.8	0.9

**Table 4:** **NUP-153 kinetic and equilibrium parameters for HIV-1 CA from clades A1, A2, B, C, and D.** Equilibrium dissociation constant (K_D_) derived from a Langmuir isotherm equilibrium fit or from a global fit and the derived kinetic data. N.B. = no binding.

HIV-1 Clade	K_D_ [μM] Equilibrium	±SD	k_on_ [M^−1^s^−1^]	±SD	k_off_ [s^−1^]	±SD	K_D_ [μM] Kinetic/Global	±SD
A1	**N.B.**	**N.B.**	**N.B.**	**N.B.**	**N.B.**	**N.B.**	**N.B.**	**N.B.**
A2	443.3	38.1	130 × 10^3^	3.75 × 10^2^	9.60 × 10^−1^	2.13 × 10^−1^	746.3	48.5
B (NL4.3)	380.7	34.6	1.57 × 10^3^	3.60 × 10^2^	7.63 × 10^−1^	7.35 × 10^−2^	499.3	94.1
C	503.0	46.9	9.33 × 10^2^	4.31 × 10^2^	7.47 × 10^−1^	7.35 × 10^−2^	881.0	267.2
D	308.7	42.8	1.89 × 10^3^	2.91 × 10^2^	6.86 × 10^−1^	1.35 × 10^−1^	367.7	83.0
